# Effect of Carbon Support, Capping Agent Amount, and Pd NPs Size for Bio-Adipic Acid Production from Muconic Acid and Sodium Muconate

**DOI:** 10.3390/nano10030505

**Published:** 2020-03-11

**Authors:** Sofia Capelli, Davide Motta, Claudio Evangelisti, Nikolaos Dimitratos, Laura Prati, Carlo Pirola, Alberto Villa

**Affiliations:** 1 Dipartimento di Chimica, Università degli Studi di Milano, via C. Golgi 19, 20133 Milan, Italy; laura.prati@unimi.it (L.P.); carlo.pirola@unimi.it (C.P.); 2 School of Chemistry, Cardiff Catalysis Institute, Cardiff University, Park Place, Cardiff CF10 3AT, UK; MottaD@cardiff.ac.uk; 3 National Council of the Research, CNR-Istituto di Chimica dei Composti Organometallici, Via G. Moruzzi 1, 20124 Pisa, Italy; Claudio.evangelisti@istm.cnr.it; 4 Dipartimento di Chimica Industriale “Toso Montanari”, Università degli Studi di Bologna, Viale Risorgimento 4, 40136 Bologna, Italy; Nikolaos.dimitratos@unibo.it

**Keywords:** adipic acid, muconic acid, liquid phase reaction, Pd NPs size effect, sol-immobilization, biomass valorization

## Abstract

The effect of support, stabilizing agent, and Pd nanoparticles (NPs) size was studied for sodium muconate and t,t-muconic acid hydrogenation to bio-adipic acid. Three different activated carbons (AC) were used (Norit, KB, and G60) and carbon morphology did not affect the substrate conversion, but it greatly influenced the adipic acid yield. 1% Pd/KB Darco catalyst, which has the highest surface area and Pd surface exposure, and the smallest NPs size displayed the highest activity. Furthermore, the effect of the amount of the protective agent was studied varying metal/protective agent weight ratios in the range of 1/0.00–1/1.20, using KB as the chosen support. For sodium muconate reduction 1% Pd/KB_1.2 catalyst gave the best results in terms of activity (0.73 s^−1^), conversion, and adipic acid yield (94.8%), while for t,t-muconic acid hydrogenation the best activity result (0.85 s^−1^) was obtained with 1% Pd/KB_0.0 catalyst. Correlating the results obtained from XPS and TEM analyses with catalytic results, we found that the amount of PVA (polyvinyl alcohol) influences mean Pd NPs size, Pd(0)/Pd(II) ratio, and Pd surface exposure. Pd(0)/Pd(II) ratio and Pd NPs size affected adipic acid yield and activity during sodium muconate hydrogenation, respectively, while adipic acid yield was related by exposed Pd amount during t,t-muconic acid hydrogenation. The synthesized catalysts showed higher activity than commercial 5% Pd/AC.

## 1. Introduction

Nowadays many companies make efforts to convert the old traditional chemical productions to a more sustainable and environmentally friendly manufacturing following the concept of sustainable process, green chemistry, and circular economy. On the basis of these concepts, the utilization of wastes coming from production scraps of wood biomass are attracting many companies, which have the possibility to create added-value products from wastes. Adipic acid (AdA) is one of the most required bulk chemicals and it is attracting the attention of industrial companies due to its versatility. AdA is mainly used for the production of polyamides (Nylon 6,6) [1], but it is also used in automotive and packaging industries [2,3]. The global AdA market size was valued at US $5.56 billion in 2016 and is expected to increase 4.7% over the forecast period 2018–2024 [4]. AdA is traditionally produced from a strong oxidation with nitric acid of KA-oil (cyclohexanol-cyclohexanone mixture) [5]. Upon reaction, nitrogen oxides are produced, which have a number of recognized ill effects on human health [6,7]. To overtake greenhouse gases problems, the synthesis of bulk chemicals from renewable sources is a key point. In this sense, research groups are developing new strategies for the production of AdA from waste wood. These new methods involve the use of biochemical processes which consist of a first fermentation step of biomass-derived platform chemicals (i.e., glucose, benzoate, and catechol) [8,9,10] using *Escherichia coli* [11] or *Saccharomyces cerevisiae* [12] to obtain muconic acid (MA). MA can be further catalytically hydrogenated to AdA by a heterogeneous catalyzed reaction in mild operating conditions [13].

One of the most important key factor of hydrogenation reactions is the catalyst. It is well known that noble metal supported catalysts are effective for reduction processes, but sometimes high pressures or temperatures are required [14,15,16]. Therefore, the design of a specific catalyst may help to increase the reaction rate, leading to high conversion and selectivity in a shorter time than a commercial catalyst and it might help to decrease working temperature and pressure. Metal loading, type of support, synthetic method, and metal particle size are the “key” parameters that affect the catalyst activity and stability. In this work sol-immobilization method was used to prepare preformed colloidal Pd nanoparticles (NPs) supported on different activated carbon (AC) materials. The research on the influence of support for the catalytic activity of metal supported catalysts has received extensive attention in the liquid-phase heterogeneous reactions. In fact, supports can be considered an important factor to influence catalytic activity in liquid-phase hydrogenation reaction, as well as they can affect the state of supported metal, mass transfer processes, and the adsorption of reagents [17,18]. Besides the support, although chemical synthesis of NPs might appear as a complex process, by understanding how nucleation and growth of particles take place, it is possible to control NPs’ shape and size. Among all the techniques, colloidal methods are well established for preparing small metal NPs with narrow particle size distribution and achieving high degree of dispersion of metal NPs. The main parameters, which affect the NPs’ synthesis, are the NPs colloidal concentration, the amount of stabilizer, the amount of reducing agent, and the reduction temperature. Considering catalytic reactions, capping agents (i.e., stabilizers) are considered to have a great impact on the performance of metal NPs due to the hindered access of the substrate on the catalyst surface and their chemical nature [19,20]. In particular, the amount of capping agent and its chemical structure might affect catalyst activity (i.e., acting as promoters) and/or change and eventually control the selectivity in liquid-phase reactions [21]. A positive effect of the presence of a stabilizer has been observed for supported Pt, Au, and Pd NPs in different hydrogenation reactions, e.g., cinnamaldehyde [22], furfural [23], and 1-epoxy-3-butene [24] hydrogenation. Finally, the thickness of the capping agent layer can influence the catalyst activity as reported for CO oxidation reaction with Pt-based catalyst [25]. Among the possible capping agents (polymer, surfactant, polar molecule, etc.), polyvinyl alcohol (PVA) represents a quite versatile protective agent that provides good metal dispersion and good NPs’ stabilization [26].

**Figure 1:**
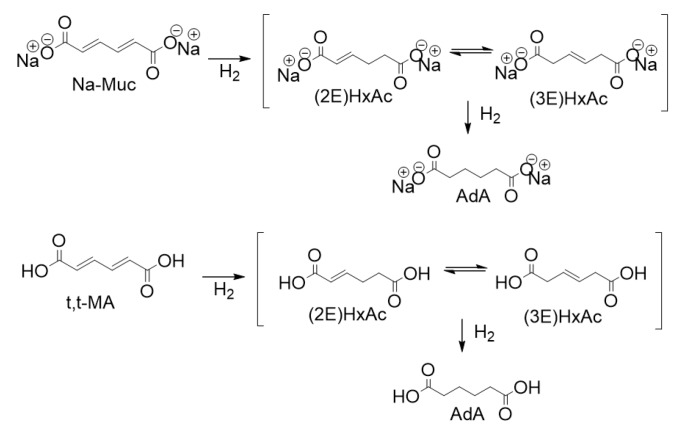
Reaction scheme of the hydrogenation of Na-Muc (top) and t,t-MA (bottom).

In this work, Pd supported NPs were used as potential catalysts due to their high activity in hydrogenation reactions [14]. Sol-immobilization method was used to synthesize a series of seven catalysts varying the AC support and the amount of PVA. Therefore, the catalytic performance of the designed catalysts was evaluated for the hydrogenation reactions of *trans,trans*-muconic acid (t,t-MA), and sodium muconate (Na-Muc) (Figure 1). During the reaction two main intermediates, (2E)-hexenedioic acid ((2E)HxAc)) and (3E)-hexenedioic acid ((3E)HxAc)), were produced. Since the operating conditions were already optimized in a previous study [27], we decided to perform the reaction using 1% Pd_PVA_/AC catalysts prepared by sol-immobilization method keeping constant the reaction temperature (50 °C), hydrogen pressure (1 bar), substrate concentration (0.014 M), and metal to substrate ratio (1/200 mol_Pd_/mol_substrate_). In this report, we show how the choice and the nature of AC support and PVA amount can affect NPs’ size and the catalyst behavior, increasing the initial activity with respect to a commercial 5% Pd/AC.

## 2. Materials and Methods

### 2.1. Catalyst Synthesis

The 1% Pd_PVA_/AC catalysts were prepared using colloidal-immobilization synthesis. The 400 mL of HPLC grade water (Fischer Scientific, Milan, Italy) were added in a 600 mL flask. The water was stirred at 800 rpm using a magnetic stirring. Then 2 mL of K_2_PdCl_4_ (5 mg_Pd_/mL) (Fisher Scientific) water solution and 0.65 mL of a water solution of polyvinyl alcohol 87–90% hydrolyzed (PVA) (1% wt) were added to water under magnetic stirring using a PVA/met ratio from 0.0 to 1.2 (wt/wt). Therefore, 0.2 M solution of NaBH_4_ was prepared weighting 0.075 g of NaBH_4_ in 10 mL of distilled water. Then 4.7 mL of the NaBH_4_ solution were added to the aqueous metal precursor solution (NaBH4/metal = 10 (mol/mol)). Then the colloidal solution was maintained under stirring for 30 min to stabilize the NPs formed during the reduction process. Activated carbon support (0.99 g) was then added and two drops of sulfuric acid (98%, Sigma Aldrich) were dropped into the colloidal solution. At low pH (acid environment) the surface of the carbon support was charged with a positive value, while the Pd–PVA complex remained negative. Therefore, there was an electrostatic attraction between the carbon and the metal NPs, which helped the anchoring of the NPs on the support. The mixture with the support was maintained under magnetic stirring for 1 h to allow a complete immobilization of the metal NPs on the carbon support. Finally, the prepared catalyst was separated from the aqueous solution by vacuum filtration. During the filtration, the catalyst was washed with 1 L of distilled water at room temperature. Then the filtered black powder was dried in oven at 100 °C for 16 h. The water recovered from the filtration step was analyzed by inductively coupled plasma (ICP) analysis and a negligible amount of Pd (<2%) was lost during the whole catalyst synthesis. Three different activated carbon supports were used: Activated carbon Darco KB 100 mesh, Norit^®^, and Darco G60 100 mesh activated charcoal provided by Sigma Aldrich. The results of these catalysts were compared with the results obtained using 5% Pd/AC commercial catalyst (Sigma Aldrich) normalizing the activity on the Pd amount.

### 2.2. Hydrogenation Reaction

A low-pressure glass reactor was designed to perform hydrogenation reaction at 1 bar and mild temperatures. The glass reactor was equipped with an external jacket linked to a thermostatic water bath set at 50 °C and with a pressure gauge for the control of the static hydrogen pressure. The glass cap of the reactor was equipped with a silicon septum that allowed us to make a withdrawal at different reaction times without stopping the reaction or losing pressure. The glass reactor was placed on a hot plate for the magnetic stirring. A known amount of reagent solution (100 mL) was placed in the reactor and it was heated at the desired temperature under magnetic stirring. Sodium muconate was prepared adding a stoichiometric amount of NaOH to t,t-MA (Sigma Aldrich, purity >98%). The reactant solutions were prepared at 0.014 M both for t,t-MA and Na-Muc. Then 1% Pd_PVA_/AC catalysts were added with a ratio 1/200 (mol_Pd_/mol_substrate_), and the reactor was pressurized at 1 bar after being purged with hydrogen 3 times. The zero time of the reaction was taken after the addition of hydrogen at the desired temperature (50 °C). The sample was collected using a syringe equipped with a needle of the proper length and then was filtered using a filter paper to remove the solid catalyst. Conversion and selectivity were evaluated as reported in Capelli et al. [13]. Briefly, after the end of the reaction, 1 g of the reaction mixture was used for UV analysis sample preparation. The analysis was performed from 500 to 190 nm using distilled water as blank. The value of absorbance of t,t-MA and Na-Muc was kept at 264 nm. The conversion was evaluated using Equation (1)

(1) $$Conversion\;\left(\%\right)=\frac{mol_{i}^{IN}-mol_{i}^{OUT}}{mol_{i}^{IN}}\cdot 100$$

where *mol^IN^_i_* are the moles of the substrate used for the reaction while *mol^OUT^_i_* are the moles of the substrate that remained after the reaction.

Yield of AdA and the intermediates were evaluated by using a gas chromatographer (GC) equipped with a fame ionization detector (FID) after products’ derivatization.

The remaining filtered liquid sample was dried in an oven at 70 °C to remove the reaction solvent; in this way a white/yellow solid product was obtained. The product was then esterified in methanol. Then, 5 mL of methanol were added to a catalytic amount of sulphuric acid. The reaction was performed at 70 °C for 48 h. The selectivity was evaluated analyzing the esterified products by gas chromatographic analysis using dimethyl glutarate as internal standard. SP-2380 capillary column (Sigma Aldrich) was used allowing the separation of the different stereoisomers in isothermal mode at 180 °C. The temperature of the injector and the detector was 220 °C. He, air, and H_2_ flows were 43 mL/min, 310 mL/min, and 38 mL/min, respectively. The injection volume was 1.5 µL and the analysis time 8 min. Selectivity and product yields (Y) were evaluated using Equations (2) and (3), respectively,

(2) $$Selectivity_{n}\left(\%\right)=\frac{mol_{n}}{mol_{n}+\sum mol_{i}}\cdot 100$$

where *mol_n_* is the number of moles of the considered reaction product and Σ*mol_i_* is the sum of the moles of all the other reaction products.

(3) $$Yield_{n}\left(\%\right)=\frac{Selectivity_{n}\left(\%\right)}{100}\cdot Conversion$$

The results were also expressed in terms of activity normalized on the number of exposed metal surface atoms. Initial activity was evaluated considering the amount of catalyst used for each test, the conversion, and the time of the reaction (Equation (4)).

(4) $$Activity\;\left(s^{-1}\right)=\frac{Conversion\;\left(\%\right)/100\cdot C_{s}^{0}\cdot V}{n_{Pd}\cdot reaction\;time\cdot 60\frac{s}{min}}$$

where *C^0^*_s_ (M) is the initial concentration of the substrate, *V* (L) is the liquid volume of the solution, *n_Pd_* (mol) are the moles of Pd introduced in the reactor, and reaction time (min) is the considered reaction time. The reactions were performed three times to evaluate the relative error of the analyses stated with the error bars. Activity_NS_ was then calculated normalizing the results of the activity on the number of Pd atoms (N_T_) that were covering the catalyst surface. The evaluation of the number of exposed Pd atoms was performed by assuming that all the NPs had cuboctahedral morphology with cubic close-packed (ccp) structure in this size range, then a full-shell NPs model was adopted [28]. The total number of Pd atoms in the cluster for a given cluster size can be calculated using the following equation:

(5) dsph=1.105·dat·NT13

where $d_{sph}$ is the mean diameter of Pd particles obtained from TEM analyses and $d_{at}$ is the atom diameter of Pd (0.274 nm). The number of surface atoms (*N_s_*) and *n* can be calculated from Equations (6) and (7), based on the values of N_T_.

(6) $$N_{T}=\frac{10n^{3}-15n^{2}+11n-3}{3}$$

(7) $$N_{s}=10n^{2}-20n+12$$

Therefore, the activity_NS_ based on the surface atoms can be calculated as follows [29]:

(8) $$A=\frac{N_{S}}{N_{T}}\cdot 100$$

(9) $$Activity_{NS}\;\left(s^{-1}\right)=\frac{Activity\;}{A}$$

where *A* is the fraction of atoms lying at the surface. A 5% Pd/AC commercial catalyst (by Sigma Aldrich) was also used to compare the results in terms of activity.

### 2.3. Catalyst Characterization

Fresh and used commercial 1% Pd_PVA_/AC catalysts were characterized using Brunauer–Emmett–Teller (BET), transmission electron microscopy (TEM), and X-ray photoelectron spectroscopy (XPS) analyses.

Surface area and porosimetry measurements were performed on a Quantachrome Autosorb, and samples were firstly degassed (120 °C, 3 h) prior to measuring. Surface area was calculated using the Brunauer–Emmett–Teller (BET) method based on adsorption data in the partial pressure (P/P_0_) range 0.05–0.35. Pore volume was calculated by a single point method from the amount of nitrogen adsorbed at P/P_0_ = 0.99.

Transmission electron microscopy (TEM) was performed on a ZEISS LIBRA200FE microscope operating at 200 kV (field emission gun source). Samples were prepared by sonication in isopropanol and deposited on 300 mesh copper grids coated with lacey carbon film. Histograms of the particle size distribution were obtained by counting on the micrographs at least 200 particles. The mean particle diameter (d_m_) was calculated by using the formula d_m_ = Σd_i_n_i_/Σn_i_ where n_i_ was the number of particles of diameter d_i_.

X-ray photoelectron spectroscopy (XPS) was performed on a Thermo Scientific K-alpha + spectrometer. The 1% Pd_PVA_/AC was analyzed using a monochromatic Al X-ray source operating at 72 W (6 mA × 12 kV), with the signal averaged over an oval-shaped area of approximately 600 × 400 microns. Data was recorded at pass energies of 150 eV for survey scans and 40 eV for high-resolution scan with a 1 eV and 0.1 eV step size, respectively. Charge neutralization of the sample was achieved using a combination of both low-energy electrons and argon ions (less than 1 eV), which gave a C (1 s) binding energy of 284.8 eV. All data were analyzed using CASAXPS (v2.3.17 PR1.1) using Scofield sensitivity factors and an energy exponent of −0.6.

## 3. Results

### 3.1. Support Effect

A series of 1% Pd_PVA_/AC catalysts were synthesized by sol-immobilization method and characterized by means of BET, transmission electron microscopy (TEM), and X-ray photoelectron spectroscopy (XPS). These catalysts were then used for catalytic hydrogenation of Na-Muc and t,t-MA at 50 °C and 1 bar of hydrogen.

Then, three different catalysts were prepared using a metal/NaBH_4_ ratio of 1/8 (mol_Pd_/mol_NaBH4_) and a metal/PVA ratio of 1/0.65 (wt_Pd_/wt_PVA_) [29,30]; these conditions allow having good NPs’ dispersion and Pd colloidal stability. We chose three different activated carbons: Norit, Darco KB 100 mesh (KB), and Darco G60 100 mesh (G60) (Table 1) due to their different physical–chemical properties like surface area, pore diameter, and type of surface functionalities.

The surface area, micropore volume, total pore volume, micropore area, and average pore radius were evaluated using BET analysis (Table 2, Figures S1–S3). KB and Norit activated carbons are microporous supports having a surface area of 1604 and 1195 m^2^/g, respectively, and a pore size of 2.0 and 5.5 nm, respectively. G60 is a mesoporous support with the lowest surface area (802 m^2^/g) and the highest pore radius (18.7 nm).

**Figure 2:**
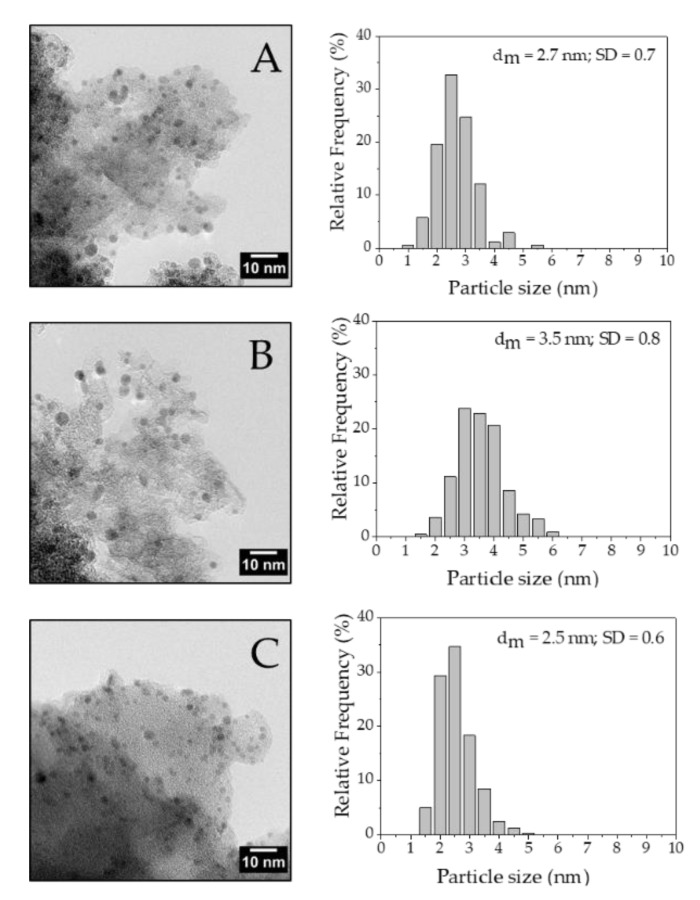
TEM of (**A**) Pd/Norit_0.65, (**B**) Pd/G60_0.65 and (**C**) Pd/KB_0.65.

TEM images of the fresh catalysts show that Pd/Norit_0.65 and Pd/KB_0.65 had comparable mean Pd particle size (2.7 and 2.5 nm, respectively), while Pd/G60_0.65 exhibited a larger mean NPs’ diameter (3.5 nm). Metal NPs were homogeneously dispersed and no big Pd particle aggregates were observed (Figure 2).

Hydrogenation reaction was performed both on Na-Muc and t,t-MA at 50 °C and 1 bar of hydrogen for a period of 90 min.

**Figure 3:**
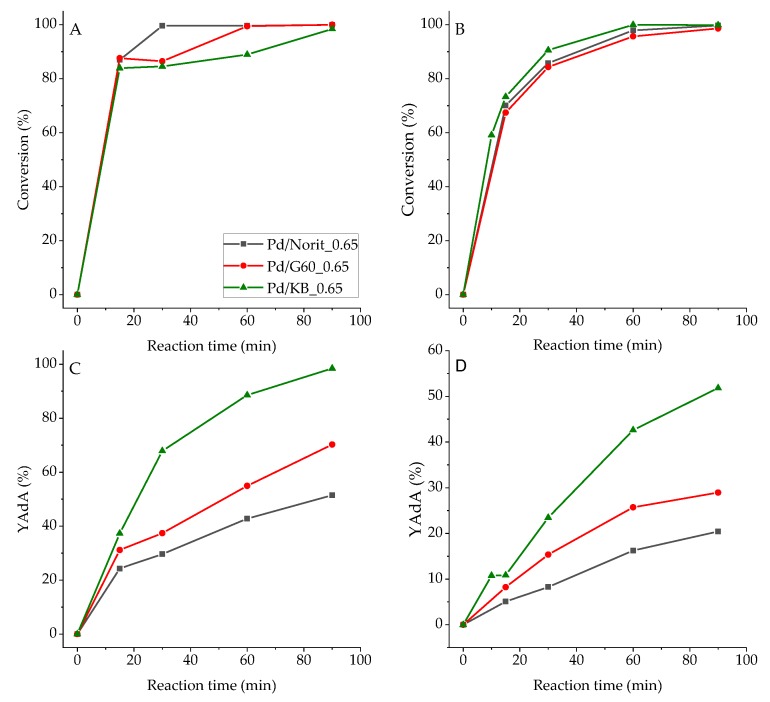
(**A**) The t,t-MA and (**B**) Na-Muc conversion, (**C**) and (**D**) AdA yield (YAdA) from t,t-MA and Na-Muc hydrogenation, respectively.

Full conversion of substrate was obtained after 90 min for both Na-Muc and t,t-MA, while the yield to AdA was different (Figure 3). In fact, Na-Muc was not completely hydrogenated to AdA; 58% of AdA yield was reached with Pd/KB_0.65 sample, while with Pd/G60_0.65 and Pd/Norit_0.65 AdA yield was 26% and 19%, respectively. On the other hand, using t,t-MA as substrate 100% of AdA, yield was obtained after 90 min with Pd/KB_0.65 catalyst. AdA yield for Pd/G60_0.65 (64%) and Pd/Norit_0.65 (46%) was higher than the one obtained during Na-Muc hydrogenation. Therefore, the physical properties of the carbon support should play a key role in the reaction activity and yield to AdA. Even though Pd/Norit_0.65 and Pd/KB_0.65 showed a similar NPs’ size distribution, the highest surface area and the lowest average pore radius of KB activated carbon facilitated the hydrogenation reaction due to a higher exposure of active sites reachable by the reagent. The Pd surface exposure (Pd/C ratio) obtained from XPS analysis (Table 4; Figures S4–S6) showed that Pd/G60_0.65 had the lowest Pd/C (0.013) and Pd/Norit_0.65 had a Pd/C ratio of 0.014, while the highest Pd surface exposure belonged to Pd/KB_0.65 sample (0.017). For all these reasons, we decided to select Darco KB AC for the synthesis of other catalysts obtained varying the amount of capping agent (PVA). In fact, the capping agent can influence the activity and yield of the desired products in the liquid phase reactions, as it was recently shown, when PVA was used for the synthesis of Pd/AC catalyst [31]. The list of the synthesized catalysts is reported in Table 1.

### 3.2. Influence of PVA Amount

The catalysts prepared with 1/0.00, 1/0.10, 1/0.30, 1/0.65, and 1/1.20 metal/PVA weight ratios were characterized by BET, TEM, and XPS analysis. BET analysis revealed that surface area was not highly affected by the amount of PVA used during the colloidal preparation method (Figure S3). Instead, micropore volume decreased from 0.53 to 0.1 cm^3^/g, while the average pore radius increased from 2.1 to 4.6 nm. On the contrary, Pd/KB_0.0 sample showed surface area value, micropore volume, and average pore radius similar to the bare carbon support. Therefore, the catalyst synthesis without PVA did not affect the physical properties of the support (Table 3).

**Figure 4:**
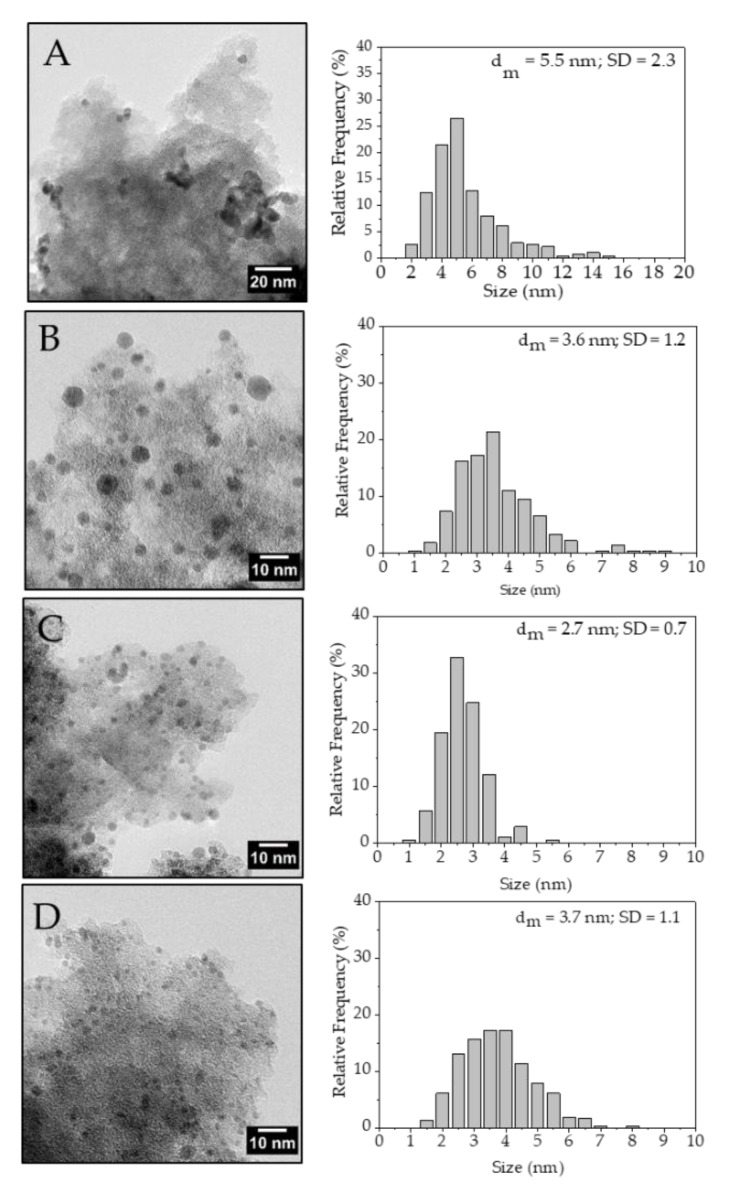
TEM of (**A**) Pd/KB_0.00, (**B**) Pd/KB_0.1, (**C**) Pd/KB_0.3, and (**D**) Pd/KB_1.2 catalysts.

TEM analysis showed that metal NPs’ size and particle size distribution were affected by the PVA amount in agreement with previous results [32] (Figure 4). Pd/KB_0.0 sample exhibited NPs with a mean diameter of 5.5 nm mainly combined to form large aggregates. Pd/KB_0.1 sample had a mean NPs’ size of 4.5 nm while Pd/KB_0.3 and Pd/KB_1.2 catalysts had very similar mean NPs’ size (3.6 and 3.7 nm, respectively). On the other hand, Pd/KB_0.65 sample had the lowest mean Pd NPs’ size (2.7 nm). In all the catalysts Pd NPs were highly dispersed on AC surface. Moreover, Pd particle size distribution was studied using TEM analysis, which was also useful to evaluate the Pd atoms number (N_S_) on the catalyst surface Equations (5)–(7) [32]. This allowed us to normalize the activity of the catalyst on the exposed Pd, excluding the effect of the particles’ size.

The catalyst surface was studied by XPS analysis (Table 4). O/C ratio increased from 0.082 to 0.150 by increasing the amount of PVA, indicating the presence of PVA on each synthesized catalyst [33]. Therefore, the PVA residues on catalyst surfaces could affect activity as well as protect Pd NPs from air passivation [29]. Pd/C ratio was also different for each sample. Pd/KB_0.1 had the highest value (0.020) while Pd/KB_0.0 the lowest one (0.007). The other catalysts showed Pd/C atomic ratio from 0.014 to 0.017 (Table 4).

**Figure 5:**
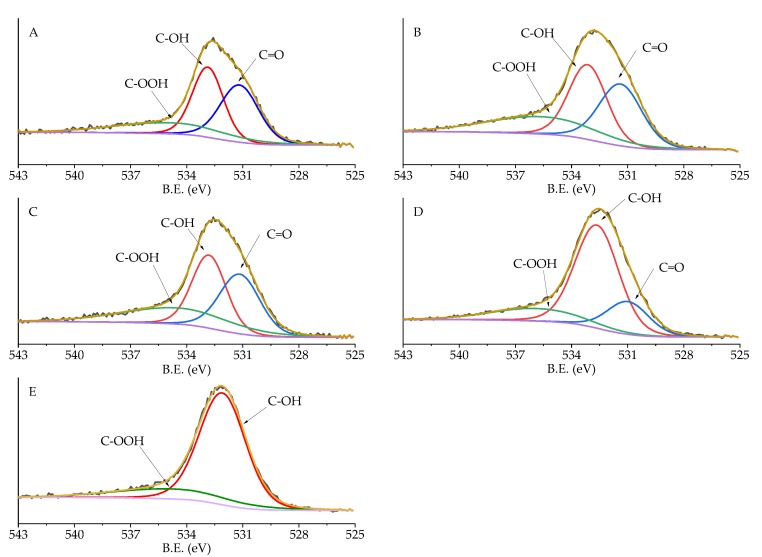
XPS spectra of O 1s region of (**A**) Pd/KB_0.0, (**B**) Pd/KB_0.1, (**C**) Pd/KB_0.3, (**D**) Pd/KB_0.65, and (**E**) Pd/KB_1.2 catalysts.

**Figure 6:**
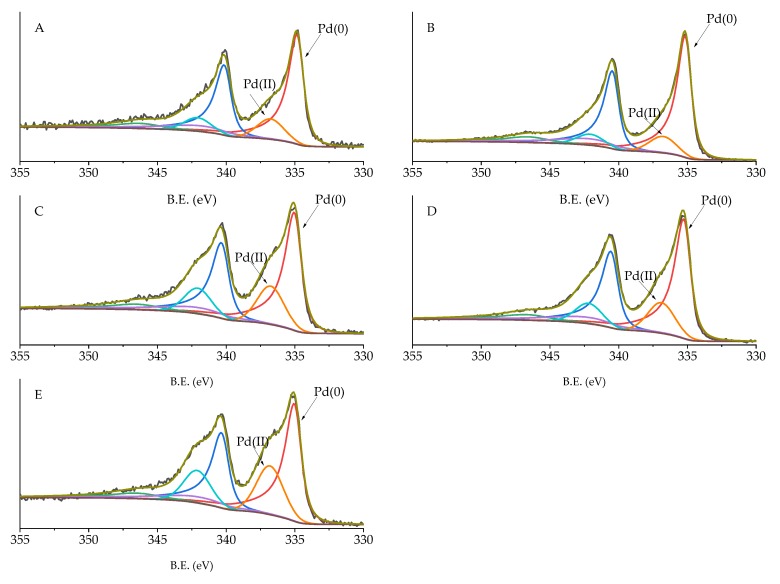
XPS of Pd 3d species for (**A**) Pd/KB_0.0, (**B**) Pd/KB_0.1, (**C**) Pd/KB_0.3, (**D**) Pd/KB_0.65, and (**E**) Pd/KB_1.2 catalysts.

High-resolution analysis of Pd 3d, C 1s, and O 1s was performed, and the obtained spectra were deconvoluted to quantify the different C and O species and Pd oxidation state (Table 5). Carbon displayed 6 different species: C sp2 (284.09–284.39 eV), C pi s (290.57–290.87 eV), C pi b (293.54–293.84 eV), C-O (287.28–289.99 eV), C=O (287.07–289.36 eV), and C sp3 (284.80 eV) [34]. No significant changes in the amount of these species were detected, except for C-O and C sp2. C-O in Pd/KB_0.1 and Pd/KB_1.2 was about 5%, which was two times higher with respect to the catalyst prepared without PVA. Considering O species (Figure 5), C=O (530.98–531.91), C-OH (532.65–532.87 eV), and COOH (534.49–535.60 eV) groups were identified [35,36]. The samples prepared with high amount of PVA (Pd/KB_0.65 and Pd/KB_1.2), which had hydroxyl groups, showed a C-OH amount higher than the ones prepared without (or with small amount) of PVA. This means that PVA was covering the catalyst surface as well as metal NPs, in agreement with previous studies [29]. The Pd_5/2_ component at about 335 eV was assigned to metallic Pd [37] and the component at 337 eV to Pd(II) [38] (Figure 6). As reported in Table 4, the catalysts prepared with high metal/PVA weight ratio showed a higher content of Pd(0) species, indicating that the presence of the stabilizer may inhibit passivation of metallic Pd surface due to air exposure [29]. This feature can have a significant impact on the catalysts’ activity and selectivity/yield, as will be discussed below since it is well known that Pd(0) is active during hydrogenation reaction. No apparent trend could be obtained for the variation of Pd(0)/Pd(II) atomic ratio according to the amount of PVA or Pd particle size despite in literature it is reported that in specific conditions it is possible to correlate Pd size with its oxidation state [39]. The same evidences were found for Ru NPs synthesized in the presence of poly(vinyl pyrrolidone) [40].

**Figure 7:**
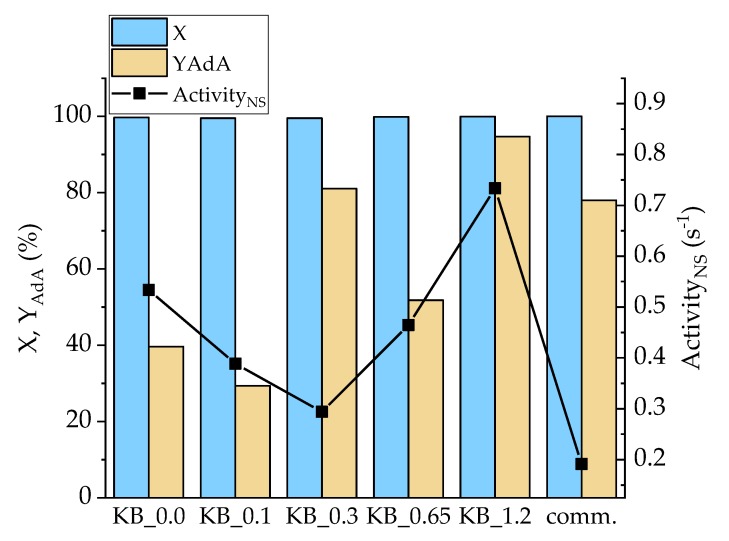
Conversion (X) and YAdA after 90 min of reaction and activity_NS_ after 15 min for Na-Muc hydrogenation.

The whole series of the synthesized catalysts were tested for Na-Muc and t,t-MA hydrogenation reaction (Figures S7–S10). After 90 min of reaction, Na-Muc was completely converted, however only with the Pd/KB_1.2 sample was AdA maximum yield (98%) reached (Figure 7). In all the cases (2E)HxAc was detected as the main intermediate (Figures S8 and S10). The catalyst activity was compared using the activity_NS_ parameter, which was the catalytic activity normalized by the number of exposed Pd atoms. This allowed us to compare catalytic results of catalysts having different mean particle size and particle size distribution. All the synthesized catalysts showed an activity_NS_ higher (>0.07 s^−1^) than the 5% Pd/AC commercial catalyst (comm.). The catalyst Pd/KB_1.2PVA had the highest yield (94.8%) and activity (0.73 s^−1^). This catalytic behavior can be explained considering that this catalyst had the highest amount of Pd(0) (32.2%), which helped the hydrogenation rate during the first minutes of reaction. Pd/KB_0.3 catalyst showed the lowest activity_NS_ (0.097 s^−1^) among the homemade catalysts. Despite the NPs’ size being similar to Pd/KB_1.2, this sample had lower Pd(0)/Pd(II) (0.37), indicating that the reaction occurred faster if metal Pd was present as the main component even if Pd NPs had the same mean size.

**Figure 8:**
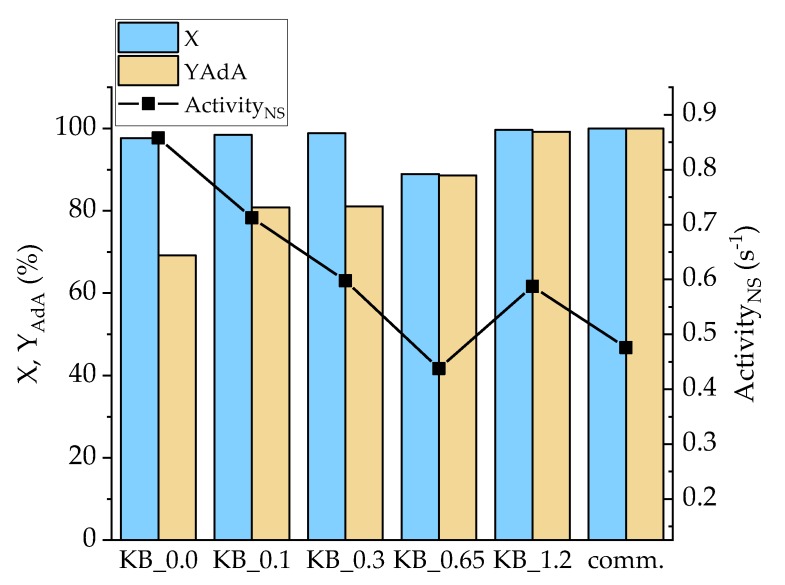
Conversion (X) and YAdA after 30 min of reaction and activity_NS_ after 15 min for Na-Muc hydrogenation.

Considering the hydrogenation of t,t-MA, all the homemade catalysts (excepted KB_0.65) showed an activity higher than the commercial 5% Pd/AC (Figure 8). The reaction after 30 min led to a full conversion of t,t-MA, while yield was in the range of 70% to 100% after 30 min of reaction. In this case, Pd/KB_1.2 catalyst showed the better results in terms of AdA yield (98%) and substrate conversion (100%). This catalyst was the one with the highest Pd(0) amount (32.2%), which helped hydrogenation reactions during the first 30 min of reactions. However, AdA yield increased by increasing the amount of PVA. Pd/KB_0.0 catalyst showed the highest activity_NS_ (0.85s^−1^) while Pd/KB_0.65 sample had the lowest value (0.43 s^−1^). Full AdA yield was obtained only for Pd/KB_1.2 and 5% Pd/AC commercial catalyst after 30 min of reaction. After 90 min of reaction, full conversion to AdA was obtained for all the catalysts presented (Figure S9).

**Figure 9:**
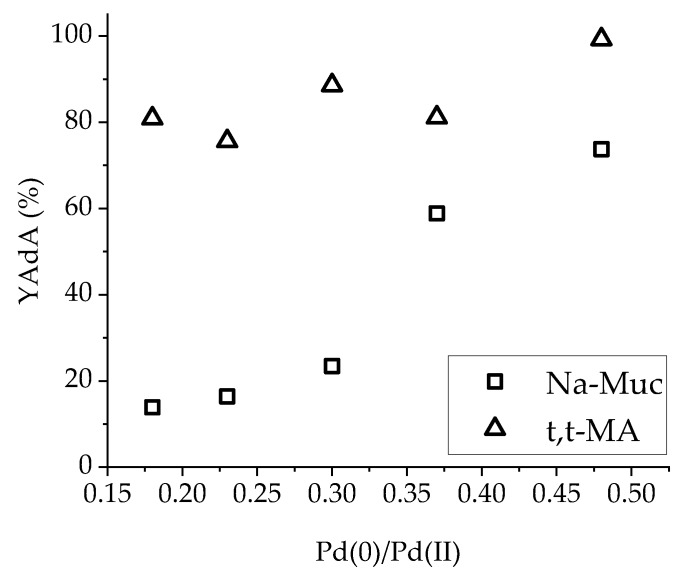
YAdA after 30 min in function of Pd(0)/Pd(II) ratio.

An upward trend was found considering AdA yield after 30 min of reaction and Pd(0)/Pd(II) ratio (Figure 9). The higher the Pd(0)/Pd(II) ratio (and, therefore, the amount of Pd(0)), the higher was the AdA yield obtained during Na-Muc hydrogenation. Otherwise, using t,t-MA as substrate, no significant changes of AdA yield were found related to the different Pd(0)/Pd(II) ratios.

**Figure 10:**
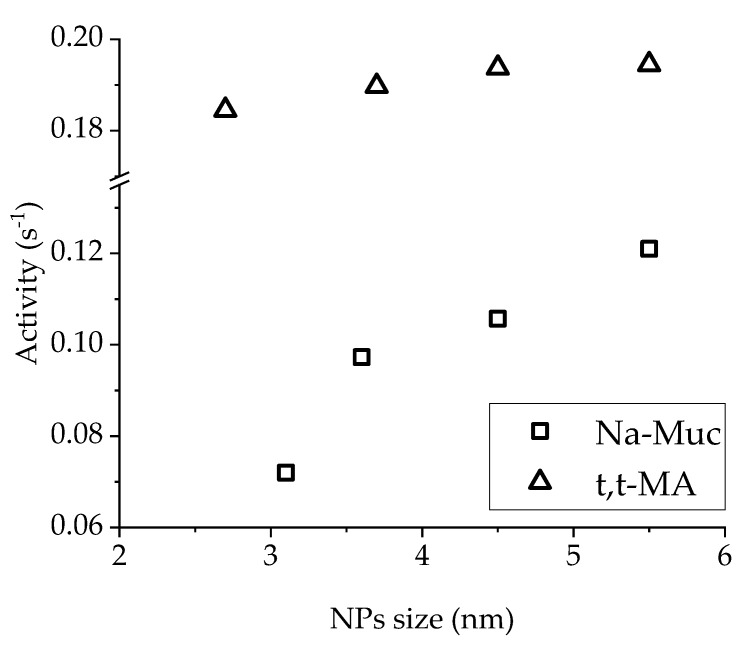
Activity (after 15 min) in function of Pd NPs size.

Considering the activity as a function of NPs’ size, a correlation was found for Na-Muc hydrogenation (Figure 10). The larger the Pd NPs in the presented particle size range (3.2–5.5 nm), the higher was the catalytic activity. For liquid-phase reaction, it is well established that smaller metal NPs give better activity [41], but in our case the gain in activity increasing Pd NPs mean size was due to the lower amount of PVA on the surface of the metal NPs. In the case of t,t-MA hydrogenation, NPs size did not significantly affect the catalytic activity.

**Figure 11:**
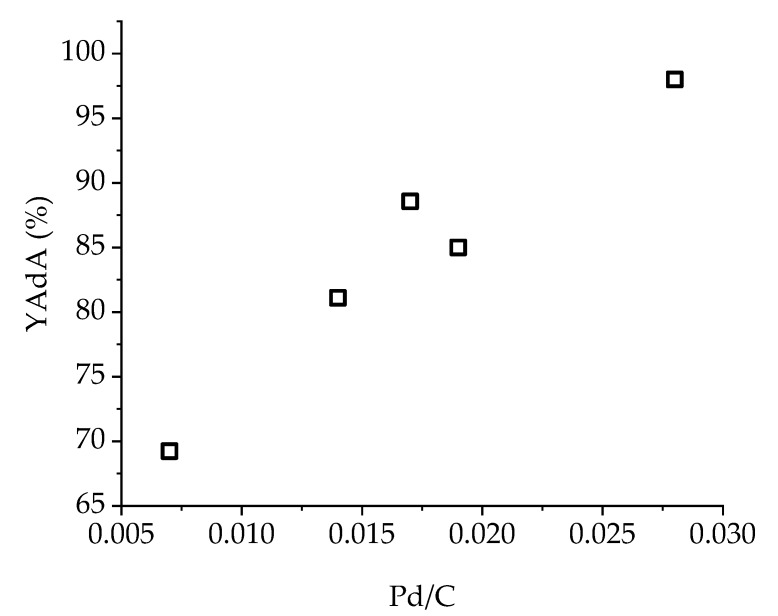
Influence of Pd/C ratio on YAdA (after 30 min of reaction) during t,t-MA hydrogenation with Pd/KB catalyst series.

XPS analysis performed on Pd/KB_0.65 catalyst showed no variation in Pd(0)/Pd(II) ratio both for Na-Muc and t,t-MA hydrogenation after 90 min of reaction. On the contrary, at the end of t,t-MA hydrogenation, the Pd/C ratio decreased from 0.18 to 0.14, revealing an increment of exposed Pd due to the removal of PVA from the surface. In fact, the C-OH group decreased from 64% to 54% (Table S1, Figure S11), indicating a partial solubilization of the capping agent in the reaction media, which was expected at the reaction temperature used for the catalytic studies. Therefore, the Pd/C ratio seemed to be the only parameter which influenced t,t-MA hydrogenation (Figure 11). The higher the amount of exposed palladium, the higher was the production and yield of AdA (after 30 min of reaction).

**Figure 12:**
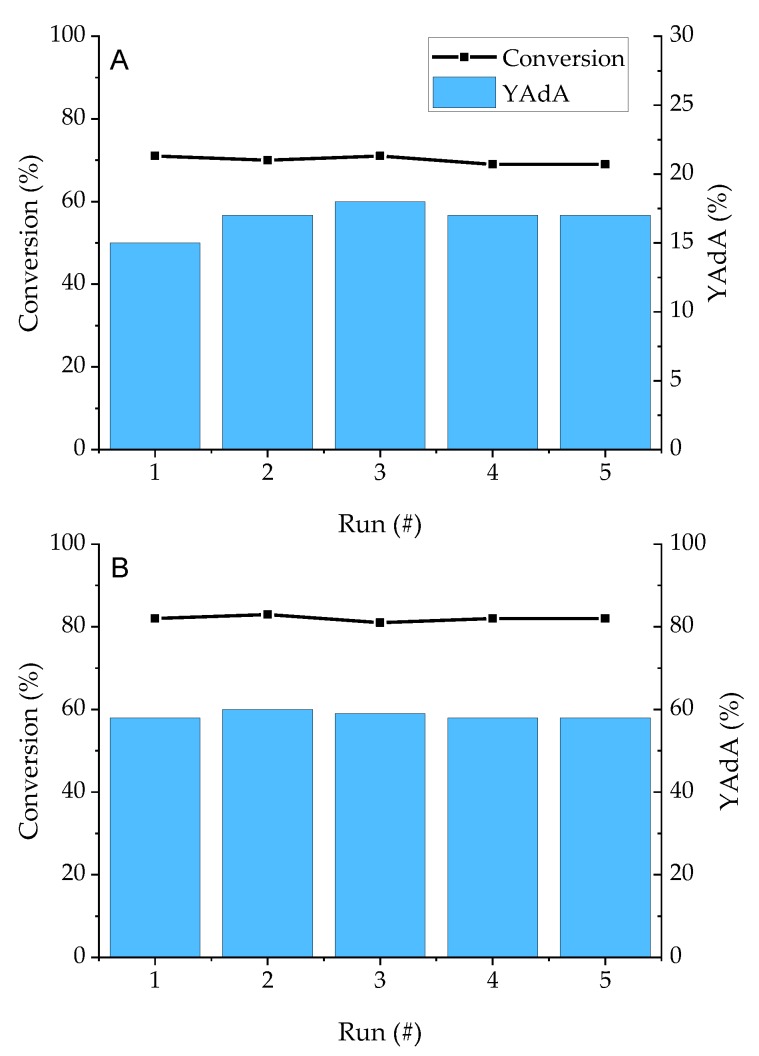
Recycling tests on (**A**) Na-Muc and (**B**) t,t-MA. Reaction time = 15 min.

Recycling tests on the Pd/KB_1.2 sample were performed to study the stability and reusability of the catalyst which gave the best results in term of conversion and AdA yield for both the reactions. Five runs were performed recovering the catalyst and without making any treatment before the reusing. Conversion and AdA yield remained constant up to 5 runs without loss in activity for both Na-Muc and t,t-MA hydrogenation (Figure 12).

## 4. Conclusions

The effect of the choice and nature of carbon support, amount of capping agent, and Pd NPs’ size was studied for Na-Muc and t,t-MA hydrogenation in mild operating conditions. Norit, Darco KB, and Darco G60 activated carbons were used to study the effect of the carbon support. Darco KB AC showed the best results in terms of activity and AdA yield due to the presence of high surface area, low average pore radius, and high surface coverage of Pd. The physical properties helped the hydrogenation reaction of both Na-Muc and t,t-MA, which led to full conversion of the substrates to AdA in 90 min. Darco KB activated carbon was then used to synthesize a series of catalysts by varying the metal/PVA weight ratio from 1/0.0 to 1/1.2. These catalysts were characterized by means of BET, TEM, and XPS analysis. Pd/KB_1.2 catalyst showed the highest activity_NS_ (0.74 s^−1^), conversion (100%), and AdA yield (97%) after 90 min during Na-Muc hydrogenation. Na-Muc reduction was significantly affected by the Pd(0)/Pd(II) ratio and the Pd NPs’ size: AdA yield was affected by the Pd(0)/Pd(II) ratio, while activity depended upon NPs’ size. The higher the Pd(0)/Pd(II) ratio and Pd NPs’ size, the higher was AdA yield and activity, respectively. All the catalyst of KB series led to full conversion of t,t-MA to AdA in 90 min. The only parameter which affected t,t-MA hydrogenation was Pd/C ratio, increasing Pd/C ratio and AdA yield. Finally, all the synthesized catalysts showed higher activity than 5% Pd/AC by a factor of at least 2 (except for Pd/KB_0.65 during t,t-MA hydrogenation). Therefore, it is possible to significantly improve the catalytic performance of hydrogenation reactions in liquid phase by tuning the support and the active phase properties. Finally, recycling tests showed the possibility to reuse the catalyst up to 5 times without losing activity.

## Figures and Tables

**Table 1:** Lists of synthesized catalyst.

Catalyst	Support	Label ^1^	Metal/NaBH_4_^2^	Metal/PVA ^3^
1%Pd_PVA_/Norit	Norit	Pd/Norit_0.65	1/8	1/0.65
1%Pd_PVA_/G60	G60	Pd/G60_0.65	1/8	1/0.65
1%Pd_PVA_/KB	KB	Pd/KB_0.65	1/8	1/0.65
1%Pd_PVA_/KB	KB	Pd/KB_0.0	1/8	1/0.0
1%Pd_PVA_/KB	KB	Pd/KB_0.1	1/8	1/0.1
1%Pd_PVA_/KB	KB	Pd/KB_0.3	1/8	1/0.3
1%Pd_PVA_/KB	KB	Pd/KB_1.2	1/8	1/1.2

^1^ Catalyst names are related to the amount of PVA (in mL) using for their synthesis. ^2^ Expressed in mol_Pd_/mol_NaBH4_, ^3^ expressed in mass_Pd_/mass_PVA_ (g/g).

**Table 2:** BET results for the bare activated carbon supports.

AC Support	Surface Area (m^2^/g)	Total Pore Volume (cm^3^/g)	Micropore Volume (cm^3^/g)	Average Pore Radius (nm)
KB	1600	1.59	0.53	2.0
Norit	1200	0.80	0.19	5.5
G60	800	0.75	0.23	18.7

**Table 3:** BET results for the bare activated carbon supports.

AC Support	Surface Area (m^2^/g)	Micropore Volume (cm^3^/g)	Average Pore Radius (nm)
Pd/KB_0.0	1600	0.48	2.0
Pd/KB_0.1	1570	0.10	4.7
Pd/KB_0.3	1550	0.10	4.6
Pd/KB_0.65	1530	0.11	4.6
Pd/KB_1.2	1490	0.11	4.6

**Table 4:** C 1s, Pd 3d, and O 1s amount obtained from survey spectra of XPS analyses.

Sample		C 1s	Pd 3d	O 1s	Pd/C	O/C
Pd/Norit_0.65	B.E. (eV)	284.07	335.08	532.07		
	A.P. ^a^ (%)	85.3	1.2	15.3	0.014	0.183
Pd/G60_0.65	B.E. (eV)	284.07	335.08	532.07		
	A.P. (%)	91.1	1.2	7.7	0.013	0.085
Pd/KB_0.0	B.E. (eV)	284.08	335.08	532.08		
	A.P. ^a^ (%)	91.9	0.6	7.5	0.007	0.082
Pd/KB_0.1	B.E. (eV)	285.08	335.08	533.08		
	A.P. ^a^ (%)	89.6	1.8	8.6	0.020	0.096
Pd/KB_0.3	B.E. (eV)	284.08	336.08	533.08		
	A.P. ^a^ (%)	88.84	1.3	9.9	0.014	0.112
Pd/KB_0.65	B.E. (eV)	285.08	336.08	533.08		
	A.P. ^a^ (%)	87.3	1.5	11.2	0.017	0.128
Pd/KB_1.2	B.E. (eV)	284.08	336.08	532.08		
	A.P. ^a^ (%)	85.98	1.11	12.90	0.013	0.150

^a^ A.P. = atomic percentage.

**Table 5:** C, Pd, and O species detected from XPS analysis.

Sample		C sp2	C pi s	C pi b	C-O	C=O	Csp3	Pd (0)	Pd (II)	Pd(0)/Pd(II)	C=O	C-OH	COOH
Pd/Norit_0.65	B.E. (eV)	284.20	-	-	288.9	286.98	285.10	335.45	337.01		532.00	533.01	535.47
	A.P. ^a^ (%)	28.0	-	-	9.9	19.2	42.9	58.5	41.5	1.41	32.2	62.7	5.1
Pd/G60_0.65	B.E. (eV)	284.30	-	-	289.10	286.57	285.21	335.5	337.4		531.89	533.34	535.58
	A.P. ^a^ (%)	54.5	-	-	5.4	13.8	23.3	52.7	47.3	1.11	20.5	58.4	21.1
Pd/KB_0.0	B.E. (eV)	284.09	290.57	293.54	287.70	287.08	284.80	334.81	336.77		531.18	532.87	534.86
	A.P. ^a^ (%)	70.6	6.3	0.5	2.2	1.7	18.8	18.5	81.5	0.23	39.4	38.6	22.0
Pd/KB_0.1	B.E. (eV)	284.39	290.87	293.84	287.28	289.36	284.80	335.13	336.77		531.38	533.13	535.60
	A.P. ^a^ (%)	63.9	5.7	0.5	4.9	2.3	22.7	15.2	84.8	0.18	35.9	39.3	25.7
Pd/KB_0.3	B.E. (eV)	294.09	290.57	293.54	288.70	287.52	284.80	335.00	336.76		531.17	532.80	534.49
	A.P. ^a^ (%)	66.1	5.9	0.4	1.1	4.2	22.2	26.9	73.1	0.37	36.3	37.9	26.8
Pd/KB_0.65	B.E. (eV)	284.29	290.78	293.75	288.99	287.19	284.80	335.23	336.89		530.98	532.65	535.87
	A.P. ^a^ (%)	63.6	5.7	0.4	2.5	4.9	22.9	23.1	76.9	0.30	19.6	64.2	16.2
Pd/KB_1.2	B.E. (eV)	284.09	290.57	293.54	286.81	288.5	284.80	335.00	336.81		-	532.81	534.60
	A.P. ^a^ (%)	66.4	5.9	0.5	4.9	2.9	19.4	32.2	67.8	0.48	-	81.2	18.7

^a^ A.P. = atomic percentage.

## Supplementary Materials

The following are available online at https://www.mdpi.com/2079-4991/10/3/505/s1: Figure S1, Adsorption isotherm performed on different AC support; Figure S2, Pore size distribution of bare activated carbon support; Figure S3, Adsorption isotherm performed on KB series catalysts; Figure S4, Deconvolution of C 1s of the (A) Pd/KB_0.65, (B) Pd/Norit_0.65, and (C) Pd/G60_0.65; Figure S5, Deconvolution of Pd 3d of the (A) Pd/KB_0.65, (B) Pd/Norit_0.65, and (C) Pd/G60_0.65; Figure S6, Deconvolution of O 1s of the (A) Pd/KB_0.65, (B) Pd/Norit_0.65, and (C) Pd/G60_0.65; Figure S7, Conversion and AdA yield (YAdA) obtained during Na-Muc hydrogenation; Figure S8, Yield of products, intermediate, and substrate during Na-Muc hydrogenation with Pd/KB_0.65 catalyst; Figure S9, Conversion and AdA yield (YAdA) obtained during t,t-MA hydrogenation; Figure S10, Yield of products, intermediate, and substrate during t,t-MA hydrogenation with Pd/KB_0.65 catalyst; Table S1, XPS results for used Pd/KB_0.65 catalyst; Figure S11, XPS of C 1s species for (A) Pd/KB_0.0, (B) Pd/KB_0.1, (C) Pd/KB_0.3, (D) Pd/KB_0.65, and (E) Pd/KB_1.2 catalysts.
