# Supplementary material for: Effect of Carbon Support, Capping Agent Amount, and Pd NPs Size for Bio-Adipic Acid Production from Muconic Acid and Sodium Muconate
Source: Nanomaterials (Basel). 2020 Mar 11;10(3):505. doi: 10.3390/nano10030505 (PMC7153248; doi:10.3390/nano10030505)
Supplement: Supplementary file 1 [file nanomaterials-10-00505-s001.pdf]

Effect of carbon support, capping agent amount and metal Pd NPs size for bio-adipic acid production from muconic acid and sodium muconate

Sofia Capelli,*[a] Davide Motta,[b] Claudio Evangelisti[c], Nikolaos Dimitratos, [d] Laura Prati, [a] Carlo Pirola, [a] Alberto Villa*[a]

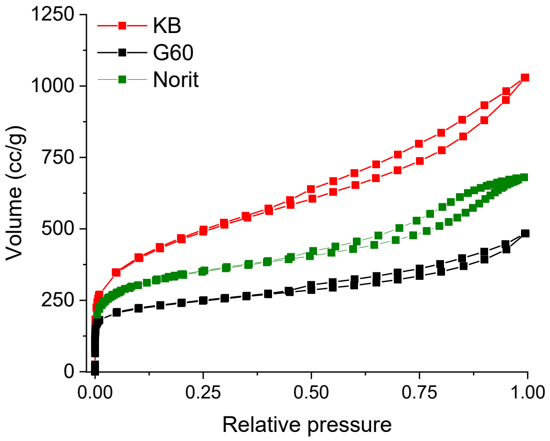

Figure S1: Adsorption isotherm performed on different AC support.

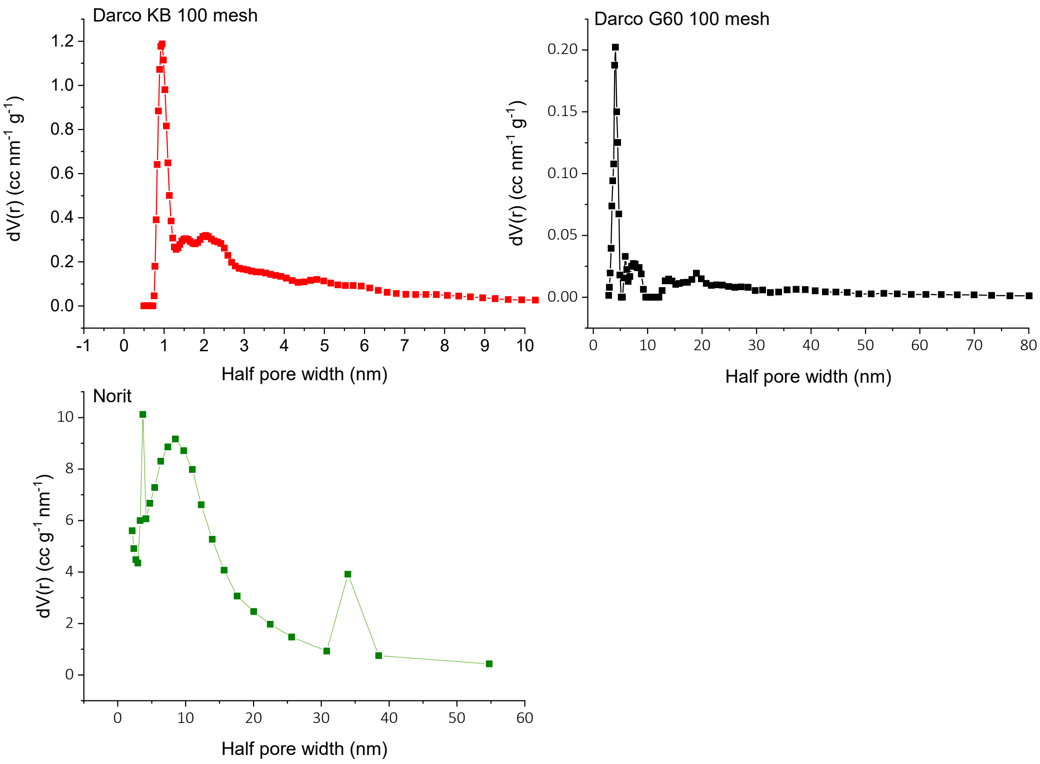

Figure S2: Pore size distribution of bare activated carbon support.

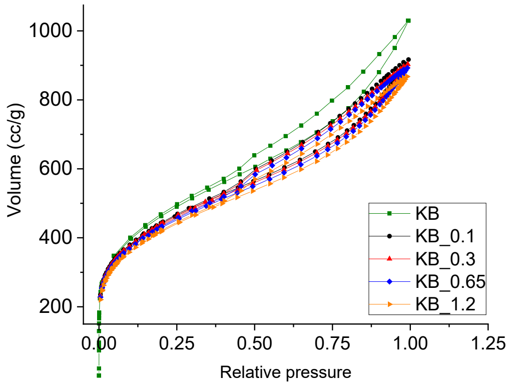

Figure S3: Adsorption isotherm performed on KB series catalysts.

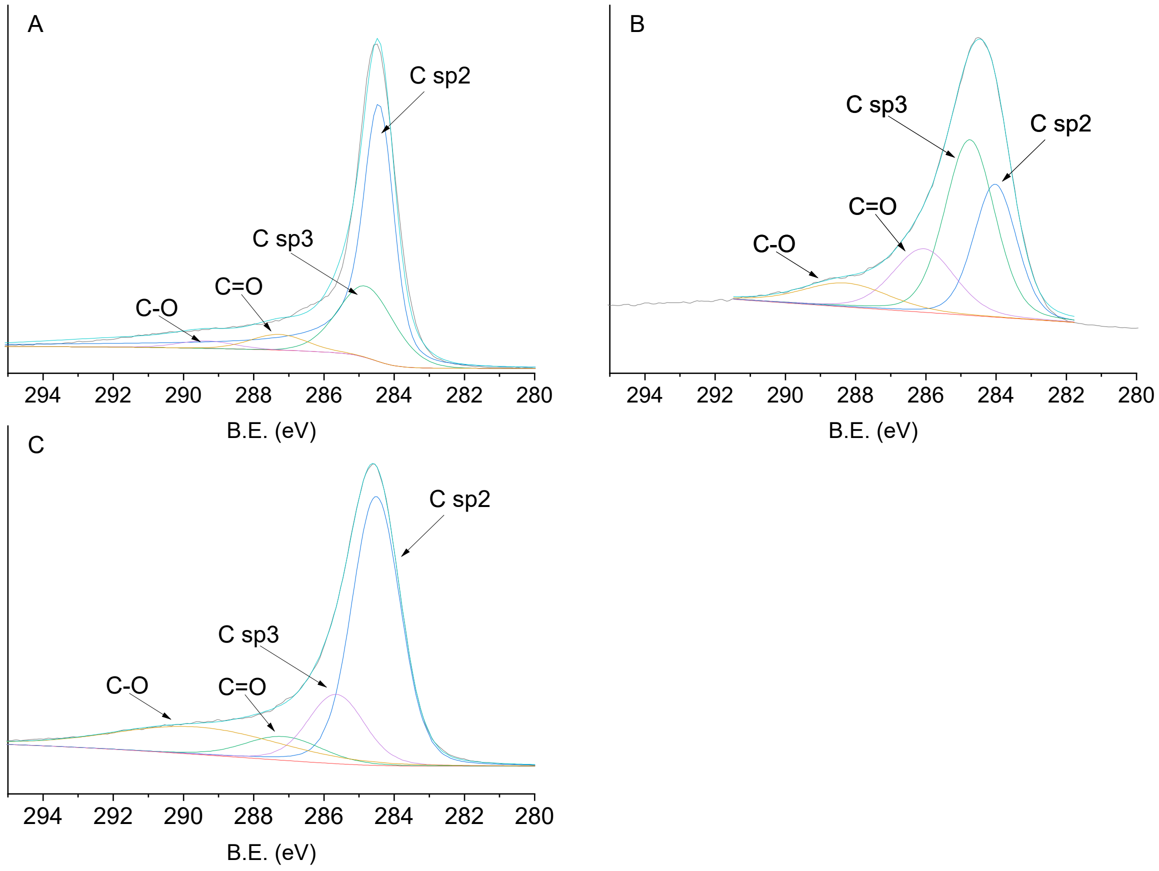

Figure S4: Deconvolution of C 1s of the A) Pd/KB_0.65, B) Pd/Norit_0.65 and C) Pd/G60_0.65.

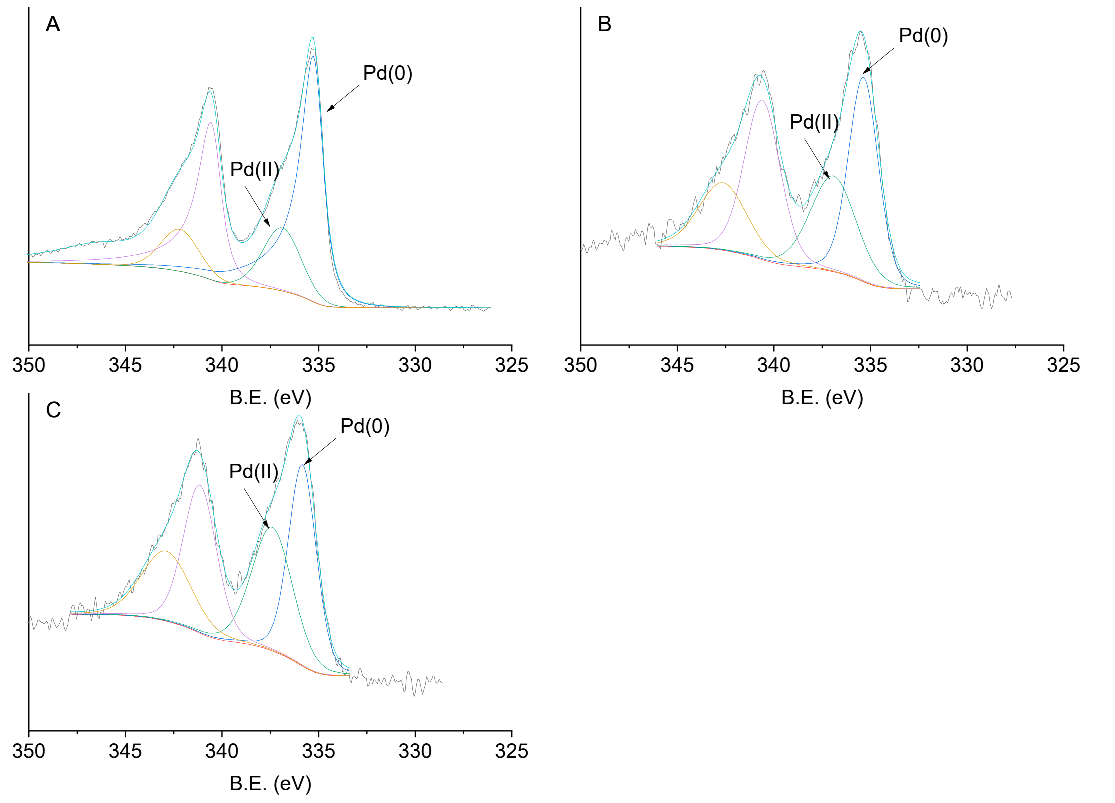

Figure S5: Deconvolution of Pd 3d of the A) Pd/KB_0.65, B) Pd/Norit_0.65 and C) Pd/G60_0.65.

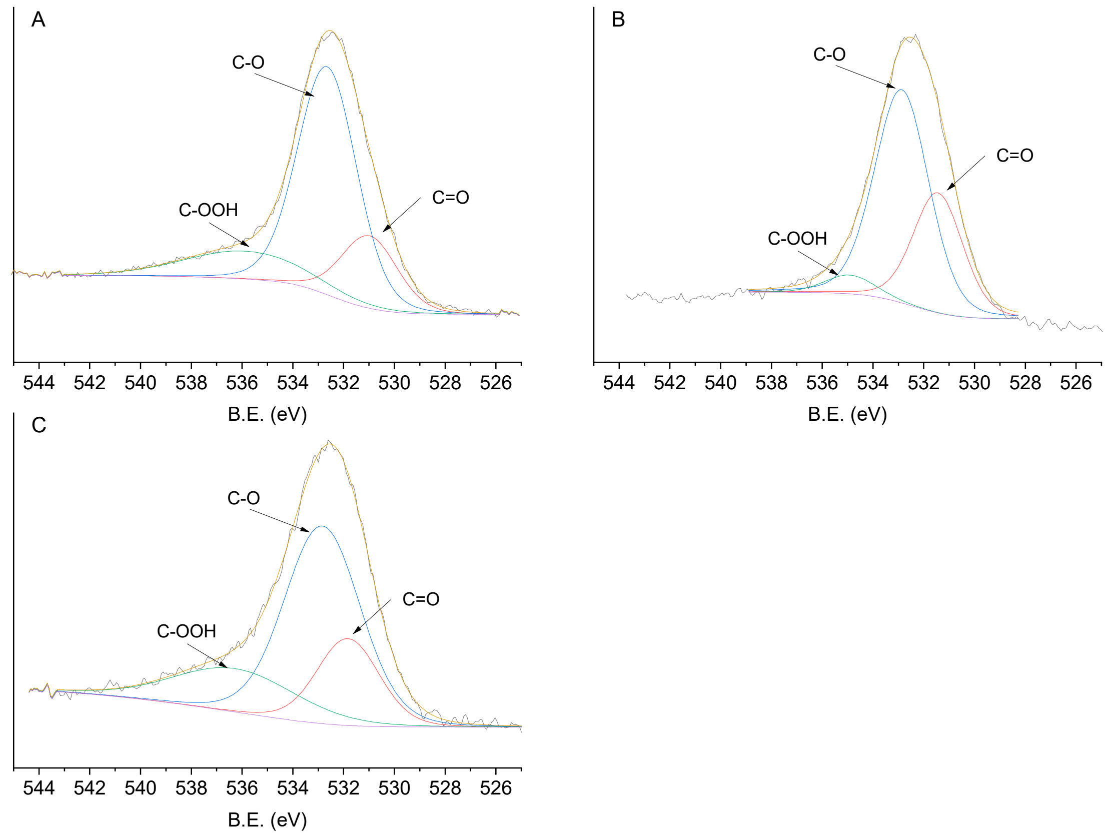

Figure S6: Deconvolution of O 1s of the A) Pd/KB_0.65, B) Pd/Norit_0.65 and C) Pd/G60_0.65.

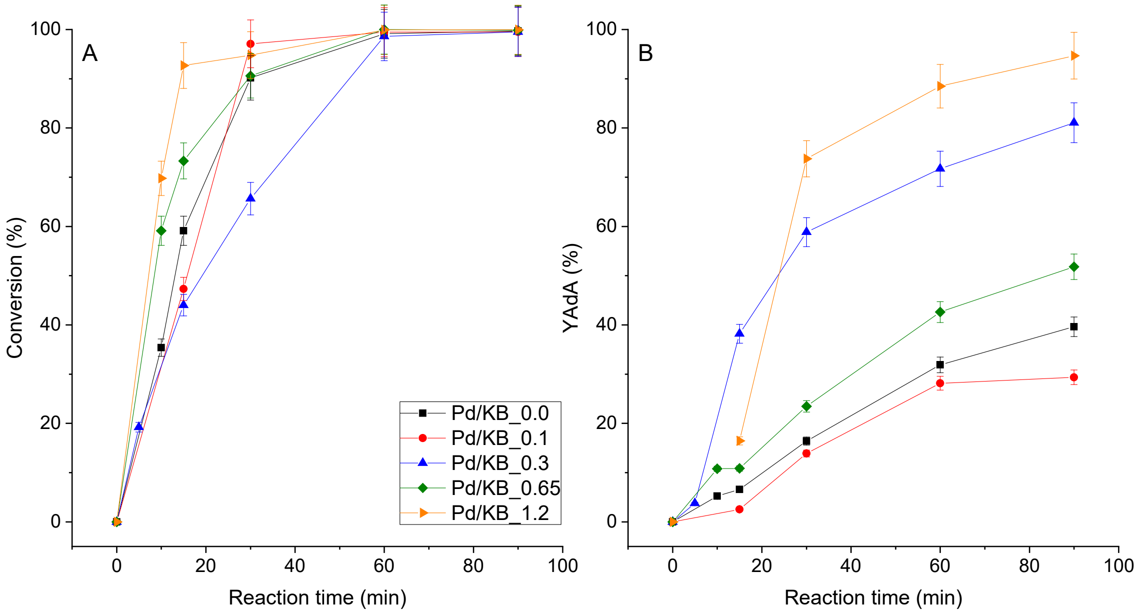

Figure S7: Conversion and AdA yield (YAdA) obtained during Na-Muc hydrogenation.

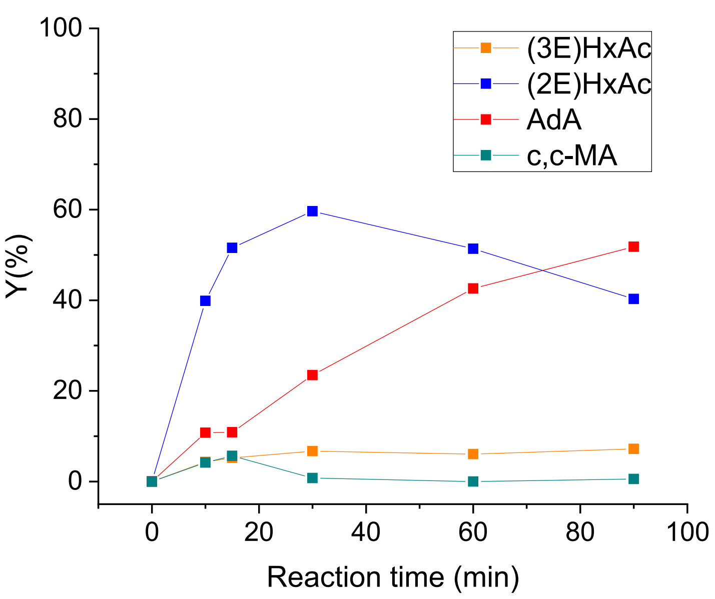

Figure S8: Yield of products, intermediate and substrate during Na-Muc hydrogenation with Pd/KB_0.65 catalyst.

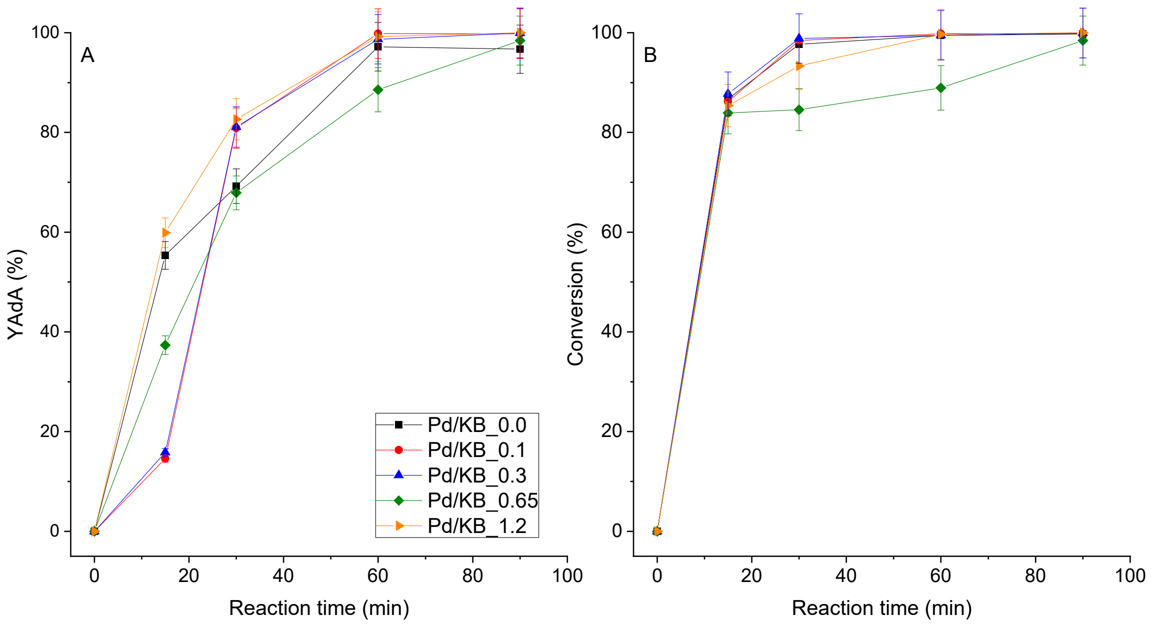

Figure S9: Conversion and AdA yield (YAdA) obtained during t,t-MA hydrogenation.

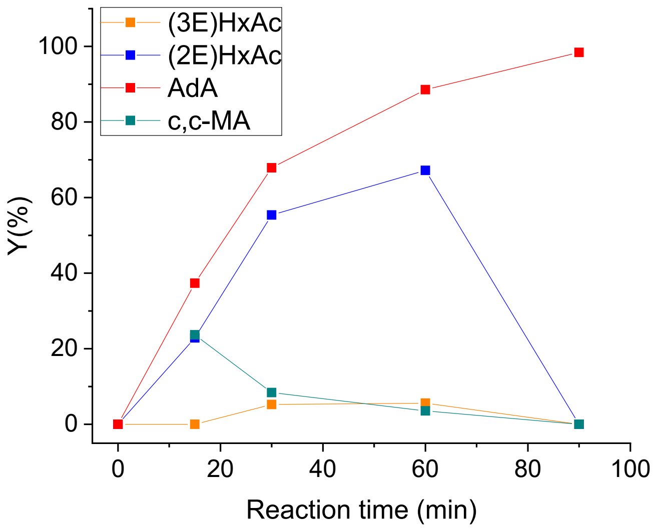

Figure S10: Yield of products, intermediate and substrate during t,t-MA hydrogenation with Pd/KB_0.65 catalyst.

Table S1: XPS results for used Pd/KB_0.65 catalyst.

Reaction time	O/C	Pd/C	Pd(0)/Pd(II)
0 min	0.13	0.017	0.30
90 min (t,t-MA)	0.17	0.014	0.38
90 min (Na-Muc)	0.20	0.019	0.34

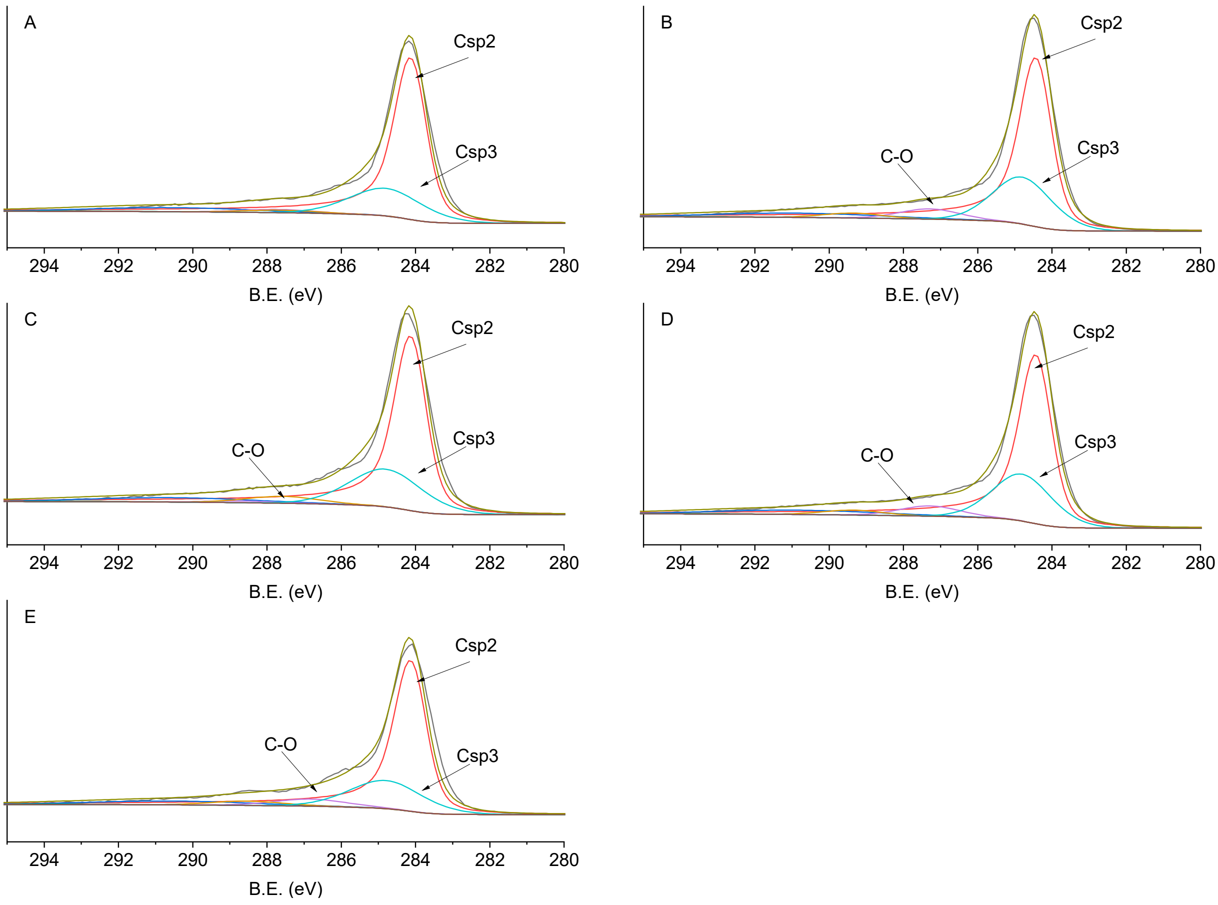

Fig S12: XPS of C 1s specie for A) Pd/KB_0.0, B) Pd/KB_0.1, C) Pd/KB_0.3, D) Pd/KB_0.65, E) Pd/KB_1.2 catalysts.
